# Functionalization of imidazole *N*-oxide: a recent discovery in organic transformations

**DOI:** 10.3762/bjoc.18.168

**Published:** 2022-11-22

**Authors:** Koustav Singha, Imran Habib, Mossaraf Hossain

**Affiliations:** 1 Synthetic Organic Research Laboratory, UGC-HRDC (Chemistry), University of North Bengal, Siliguri, Darjeeling, 734013, India

**Keywords:** functionalization, imidazole *N-*oxide, mechanistic insights, multicomponent reaction, nucleophilic substitution reaction

## Abstract

Nowadays heterocyclic compounds are widely used in medicinal chemistry and industry to develop life-saving drugs and medicines. Imidazole is one of the pharmacologically important heterocyclic motifs found in widely used and well-known medicines and bioactive molecules. The applications of imidazole derivatives displaying various biological activities, motivated researchers for the development of more potent and significant drugs containing imidazole moieties. The formation of imidazole derivatives can be achieved using imidazole *N-*oxide as starting material. In this review, the scope of substrates and reaction mechanisms of various synthetic approaches using imidazole *N-*oxides as substrates are summarized so that the chemists, researchers, and pharmaceutical industries find its effectiveness in near future for the synthesis of potent, novel, and non-toxic drug molecules.

## Introduction

**Figure 1:**
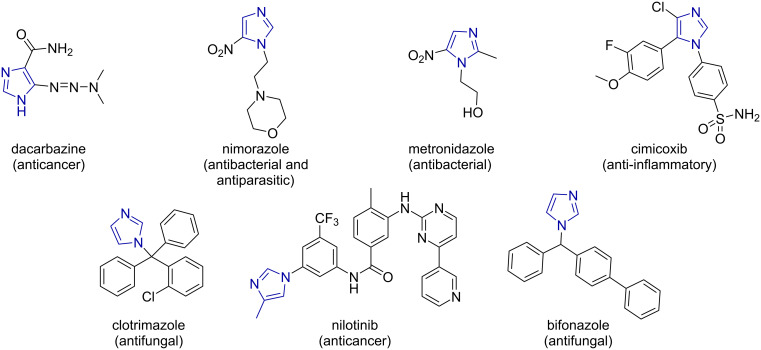
Selected imidazole-based bioactive molecules.

Imidazole is one of the best-known heterocyclic compounds. Derivatives of imidazole are powerful molecules taking part in numerous biochemical processes and exhibiting distinctive biological activities [1–2]. The imidazole motif can be seen in several natural compounds like histidine and the related local-immune hormone histamine. In the structural view of DNA and RNA, purine bases contain imidazole moieties. Also, imidazole *N-*oxides have various and intriguing applications in natural products synthesis, catalysis, and coordination chemistry [3]. Derivatives of imidazole compounds exhibit various bioactivities, such as antimicrobial [4], antidiabetic [5], antiviral [6], antihypertensive, anticancer [7], anti-inflammatory [8], analgesic [9], antimicrobial and so on (Figure 1). For the synthesis of imidazole derivatives, there are several interesting methodologies available, like cycloaddition, metal-free coupling reactions, three-component or multicomponent reactions etc., which use *N-*oxide chemistry. In 2021 Timofey D. Moseev and co-workers unfolded an attractive procedure of the coupling reaction between 2*H*-imidazole 1-oxides and polyphenols under metal-free conditions through C–H/C–H coupling [10]. Also, in 2020, Valery P. Perevalov and team reported a three-component reaction of 2-unsubstituted imidazole *N*-oxides, arylglyoxals, and CH-acids [11]. However, the use of imidazole 1-oxides is not much unfolded till now among the science community. There were quite some excellent works using imidazole *N-*oxide as starting material in recent years and the increasing number of reports proves the promising future of this field. In the past years, various excellent review papers were reported about heterocyclic *N-*oxides. For example, in 2014, Guobing Yan et al. published a review paper exploring several procedures of C–H bond activation for the functionalization of *N-*oxides [12] and in 2019, Dongli Li and co-workers analyzed heterocyclic *N*-oxides with regard to their usefulness in synthesis of organic drug molecules and catalysis [13]. Many review papers have been published regarding the synthesis of imidazole derivatives, such as the synthesis of medicinal compounds containing imidazole moieties have been reviewed by Xunan Zheng and co-workers in 2020 [14], the catalytic preparations of derivatives of imidazole were reviewed by Dr. Shin Kamijo et al. in 2007 [15] and so on. However, no such review paper related to the imidazole *N-*oxides was published till date. This is the first review work, where various reported reaction procedures of imidazole *N-*oxides are accumulated. In this review, we discuss about the utilization of imidazole *N-*oxides as precursors to form imidazole derivatives in the last few years (2015–2021), so that the researchers and chemists get interests to work using imidazole *N-*oxide and make it prosper with their fruitful efforts in future.

### Scope of the review

In this review, several reaction procedures of imidazole *N-*oxides reported in the past years are discussed. This review contains cycloaddition reactions, nucleophilic halogenation reactions, metal-free coupling reactions, formation of *N*-heterocyclic carbenes, and multicomponent reactions, mainly three-component reactions. The mentioned methodologies have been performed by either one-pot or multistep reaction processes. Here, substrate scope and proposed mechanistic pathways of the different methodologies are summarized in detail. We hope that this review will provide researchers and scientists an idea on the works related to imidazole *N-*oxides currently going on and to do more research using imidazole *N-*oxides as precursors to make this research field flourishing in near future.

## Review

### Cycloaddition reactions

**Scheme 1:**
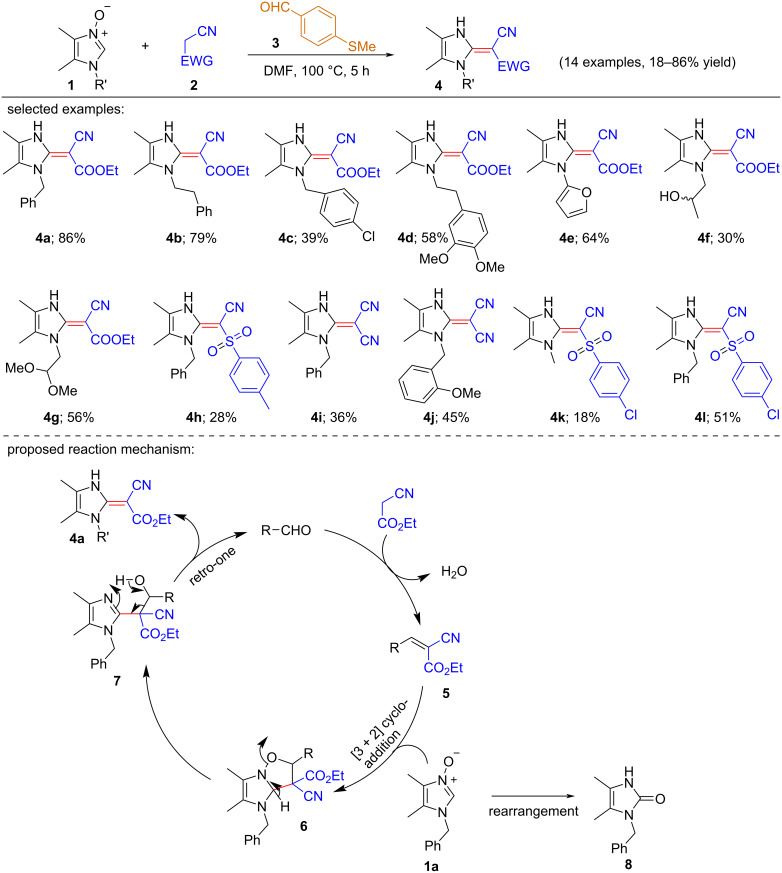
Formation of ethyl 2-cyano-2-(1,3-dihydro-2*H*-imidazole-2-ylidene)acetate derivatives via [3 + 2] cycloaddition reactions.

In 2020, Anton V. Kutasevich et al. unfolded a very interesting deoxygenative reaction procedure where 2-unsubstituted imidazole *N-*oxides reacted with ethyl cyanoacetate to generate ethyl 2-cyano-2-(1,3-dihydro-2*H*-imidazole-2-ylidene)acetates in the presence of aldehyde catalyst through a [3 + 2] cycloaddition [16]. Here, imidazole *N-*oxides behaved as 1,3-dipoles and nucleophiles in Michael-type addition reactions. The optimized reaction conditions were estimated to be a 1:1:1 ratio of 2-unsubstituted imidazole *N-*oxides as *C*-nucleophile, ethyl cyanoacetate as C–H acidic electrophile and 4-(methylsulfanyl)benzaldehyde as aldehyde catalyst, DMF as solvent at 100 °C for 5 h. Under the optimized conditions, malononitrile providing the products **4i**,**j** (36–45%), 2-[(4-chlorophenyl)sulfonyl]acetonitrile gave the corresponding products **4k**,**l** (18–51%) and 2-tosylacetonitrile producing the expected product **4h** (28%) can also act as the C–H acids in place of ethyl cyanoacetate. But the yields of the products were relatively low. Here, the product **4c** was obtained only in 39% yield because of steric effects and the electron-withdrawing nature of the substituents on the ‘N’-atom of the imidazole *N-*oxides at C-3 position (Scheme 1). Also, with the increase in chain length, the decrease in yield of products **4a**,**b** was observed. After analyzing X-ray crystallography data, the authors revealed the product **4a** as *E*-isomer due to the strong intramolecular H-bonding. It was also observed that the labile acetal group in the product **4g** remained unaffected during the reaction process under the standard conditions. Imidazole *N-*oxides reacted with ethyl cyanoacetate to produce the corresponding products **4a**–**g** in 30–86% yield regardless of electron-withdrawing and electron-donating groups. Various N-substituted imidazole *N-*oxides were screened and the yields of products were 18–86%. It was detected by NMR studies that the final expected products are found as single isomers. Based on some experimental results, a suitable mechanistic pathway has been proposed (Scheme 1). At first, imidazole *N-*oxide underwent a [3 + 2] cycloaddition reaction with ylidene **5** to form unstable cycloadduct **6**, which then rearomatized followed by ring opening to provide the intermediate **7**. Finally, the breaking of the C–C bond of intermediate **7** led to the generation of the expected final product **4a** through retro-one reaction. It was also shown that the side product, 1-benzyl-4,5-dimethyl-1,3-dihydro-2*H*-imidazol-2-one (**8**) was formed from **1a** through simply thermal rearrangement.

### Nucleophilic substitution reactions

#### Metal-free coupling reaction through nucleophilic substitution of H-atom (S_N_^H^)

**Scheme 2:**
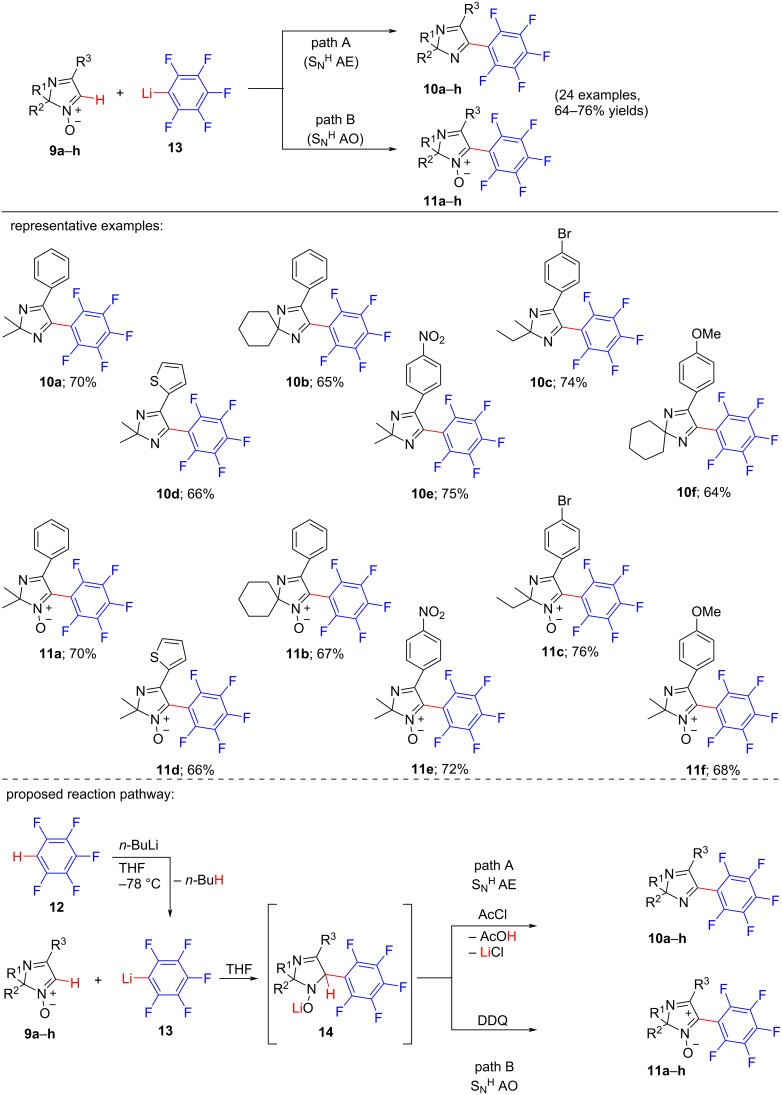
C–H/C–Li coupling reaction of 2*H*-imidazole 1-oxides with pentafluorophenyllithium.

In 2020, a C–H/C–Li coupling reaction between 2*H*-imidazole 1-oxides and pentafluorophenyllithium under transition metal-free conditions was reported by Timofey D. Moseev and co-workers [17]. The reaction proceeds through the nucleophilic substitution of a H-atom (S_N_^H^) at the C-5 position of the imidazole *N-*oxides. This reaction methodology can be considered as suitable process for the syntheses of bioactive imidazole compounds containing (poly)fluoroaryl moieties. Interestingly, in some of the perfluoroaryl-substituted 2*H*-imidazole products, a push–pull fluorophore system was discovered, which can be useful for the preparation of fluorometric sensor materials because of the solvent-dependent intramolecular charge transfer effect (ICT). Here, two reaction pathways were designed: (A) addition–elimination pathway (S_N_^H^AE), where deoxygenation of imidazole 1-oxide resulted in the formation of perfluoroarylated 2*H*-imidazoles; and (B) addition–oxidation pathway (S_N_^H^AO), where without affecting the N–O bond of imidazole 1-oxides, perfluoroarylated 2*H*-imidazole 1-oxides were obtained as final products. The study of the optimization conditions showed that the highest yields of products were obtained when: (I) in case of path A, 1.0 mmol of the 2,2-dimethyl-4-phenyl-2*H*-imidazole 1-oxide (**9a**) was reacted with pentafluorophenyllithium (**13**) (which was formed in situ from pentafluorobenzene (**12**)] in the presence of 1.0 mmol AcCl as eliminating agent (EA) in dry THF as solvent at −78 °C; (II) in case of path B, the mixture of 1.0 mmol of 2,2-dimethyl-4-phenyl-2*H*-imidazole 1-oxide (**9a**) and 1.0 mmol of pentafluorobenzene (**12**), 1.1 mmol of *n*-BuLi as base, 1.5 equiv DDQ as oxidant in dry THF as solvent was refluxed for 4 h. The screening of several 2*H*-imidazole 1-oxides revealed that the derivatives of 2*H*-imidazole 1-oxide reacted with pentafluorophenyllithium to provide the expected products in good yields (64–76%) regardless of the presence of either electron-withdrawing or electron-donating groups at the vicinal position of 2*H*-imidazole and the addition–elimination pathway providing the products **10a**–**h** or addition–oxidation pathway affording the corresponding products **11a**–**h** (Scheme 2). At first, pentafluorophenyllithium (**13**) which was produced through the reaction between *n*-BuLi and pentafluorobenzene (**12**), acted as the nucleophile to attack the C-5 position of 2*H*-imidazole 1-oxides **9a**–**h** to form the σH-adduct **14**. The use of a deoxygenation agent in the mixture led to the formation of the desired products **10a**–**h** via “addition–elimination” (S_N_^H^ AE, path A) from the adduct **14** with the elimination of the good leaving groups. The eliminating agent (AcCl) led to O-acylation of the intermediate **14** and resulted in deoxygenation through the release of AcOH giving 2*H*-imidazole derivatives. On the other hand, in case of “addition–oxidation” (S_N_^H^ AO, path B), the oxidant DDQ picked up a proton from intermediate **14** to provide the targeted products **11a**–**h**.

**Scheme 3:**
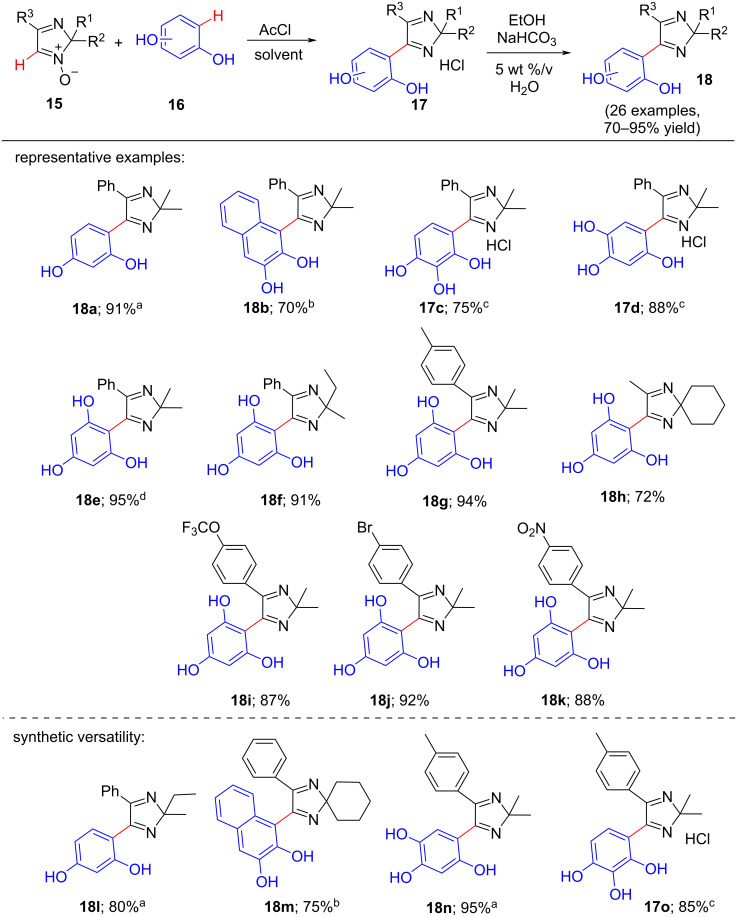
Transition-metal-free coupling reaction of 2*H*-imidazole 1-oxides with polyphenols. Reaction conditions: 1 mmol of each substrate at 0 °C to rt for 30 min; ^a^toluene and acetone mixture (2:1) as solvent; ^b^chlorobenzene and acetone mixture (2:1) as solvent; ^c^hexachloroacetone and acetone mixture (7:1) as solvent. The isolated yields are given.

In the next year (2021), the same authors suggested another fascinating deoxygenative transition-metal-free S_N_^H^ coupling reaction procedure of 2*H*-imidazole 1-oxides with polyphenols [10]. This process is convenient for producing bioactive bifunctional compounds containing both imidazole and phenolic moieties under mild conditions with two successive steps. The final products were obtained in good to excellent yields (70–95%). However, this methodology can be successfully used only for the polyphenols with at least two OH groups in one ring and is not applicable for monohydroxy group systems, which is the drawback of this method. The reaction conditions were optimized as 2,2-dimethyl-4-phenyl-2*H*-imidazole 1-oxide (**15a**) and resorcinol (**16a**) (1:1), AcCl (1.0 equiv) as activating agent at 0 °C to rt for 0.5 h. The solvents used in this reaction were acetone, a mixture of toluene and acetone (2:1), mixture of chlorobenzene and acetone (2:1), or a mixture of hexachloroacetone and acetone (7:1). Under the optimized conditions, the screening of various polyphenols showed that the yields of products were 70–95% when the mixture of toluene and acetone (2:1) was used as solvent (Scheme 3). Five polyphenols with at least two OH groups gave the corresponding products in 70–95% yields. After that, through the study of the scope of nitrones against phloroglucinol (**16e**), it was found that the products corresponding to 2*H*-imidazole 1-oxides bearing either electron-donating groups (**18e**–**h**) or electron-withdrawing groups (**18i**–**k**) present at both C-2 and C-4 positions were obtained in 72–95% yields (9 examples). It was also shown the versatility of this synthetic approach by providing various examples, few of them such as **18l**–**o** reflected as good to excellent yields (75–95%).

### Nucleophilic halogenation reactions

**Scheme 4:**
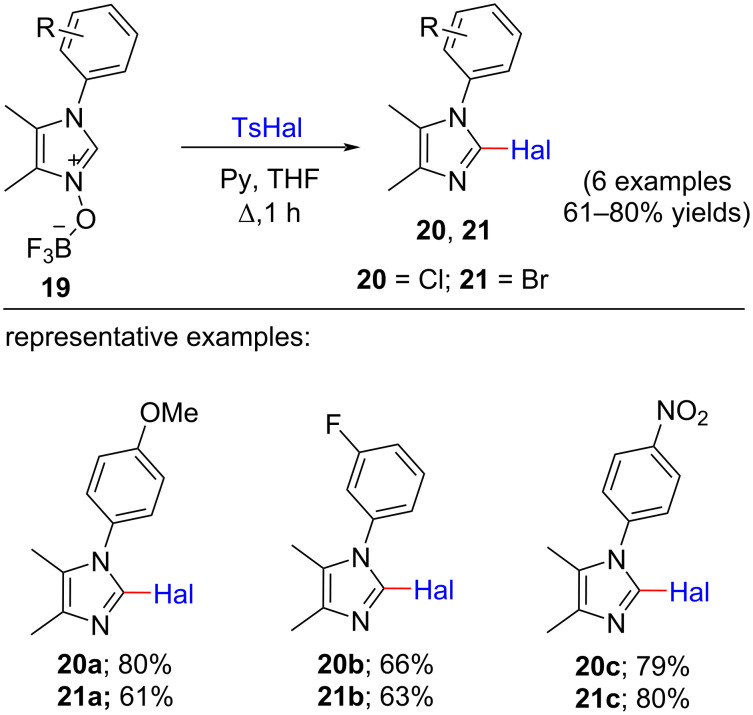
Halogenation reaction of 2-unsubstituted imidazole *N-*oxides using tosyl halogenides.

In 2015, Evgeny I. Adiulin and co-workers reported a methodology for the deoxygenative nucleophilic halogenation of 2-unsubstituted imidazole *N-*oxides at the C-2 position using tosyl halogenides (TsHal) as halogen donor in THF as the solvent [18]. In this procedure, the fluoroborate complexes of the *N-*oxides *O*-acylated by TsHal and the *cine*-substitution occurs with the help of a halogen group releasing the tosyl group. Here, 1.0 equiv 2-unsubstituted imidazole *N-*oxides were refluxed with 1.0 equiv TsHal in 1.0 equiv pyridine that played the role as both the base and the nucleophilic acylation catalyst in THF as solvent for 1 h. In this reaction, the yield for the transformation of 2-chloro-1-(4-methoxyphenyl)-4,5-dimethyl-1*H*-imidazole (**19a**) was 80% when 1.0 equiv TsCl was used. 2-Chloro-1*H*-imidazoles **20b**,**c** and the corresponding brominated products **21a**–**c** using TsBr were also successfully obtained from the reaction medium (Scheme 4). The proposed mechanism proceeded via *cine*-substitution. The rate-determining step was found to be the nucleophilic attack of the halide ion at the C-2 position of the *O*-tosylated imidazole 1-oxide. In spite of the difference in rate for the cases of chloride and bromide where bromination occurred at higher rate, *N*-tosylpyridinium halide was the acylating agent in both cases.

**Scheme 5:**
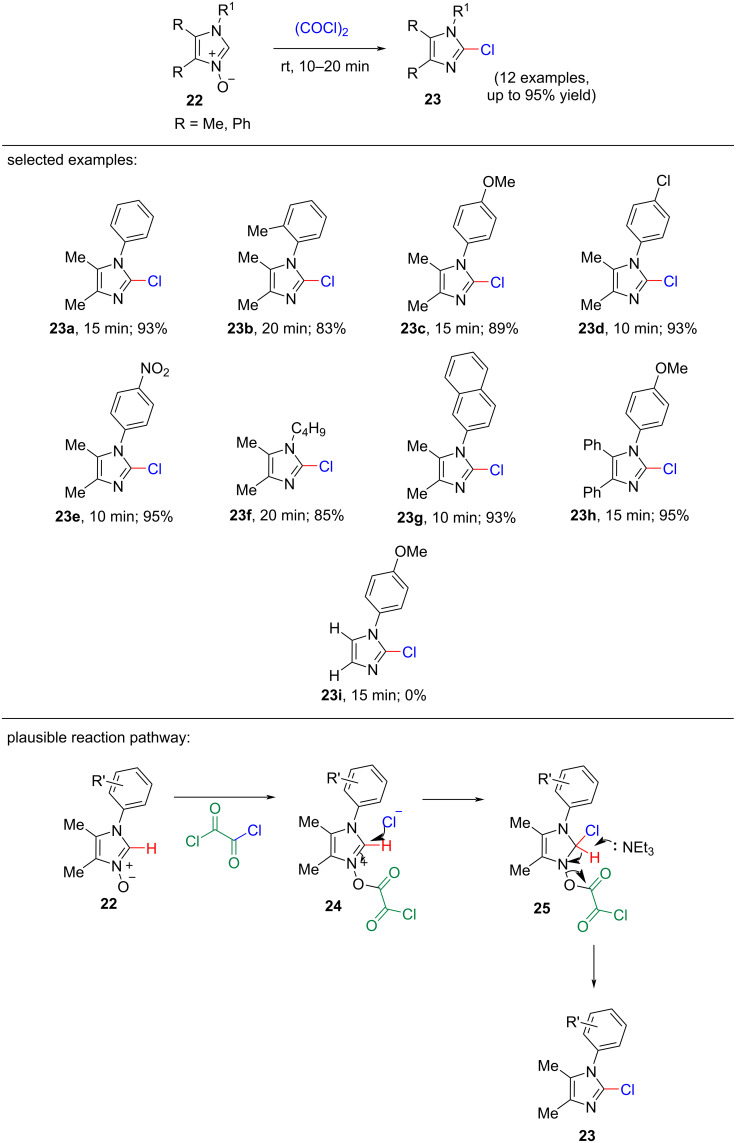
Solvent-free chlorination reaction of imidazole *N*-oxides.

In 2017, M. Hossain and co-workers suggested a quite unique one-pot deoxygenative chlorination reaction procedure of 2-unsubstituted imidazole *N-*oxides using oxalyl chloride as chlorinating agent to synthesize pharmacologically important 2-chloroimidazoles under solvent- and metal-free conditions [19]. This reaction process is expeditious and proceeds at room temperature providing excellent yields of the products with a wide substrate scope. The optimized conditions were found to be 1.0 mmol of imidazole *N-*oxide as substrate, 2.0 equiv of oxalyl chloride as chlorinating agent, 1.5 equiv of triethylamine as base at rt for only 10 minutes. Under the optimized conditions, the screening of several imidazole *N-*oxides showed that in most of the cases, the products were obtained in excellent yields (83–95%, Scheme 5). The derivatives of 2-unsubstituted imidazole 1-oxide bearing electron-withdrawing groups **23d**,**e** (93–95%) present at the C-4 position in the phenyl ring gave higher yields of products than those containing electron-donating groups **23b**,**c** (83–89%), because of the increased electrophilicity in case of electron-withdrawing groups. Imidazole *N-*oxides bearing naphthyl (**23g**) and *n*-butyl (**23f**) groups at the N-1 position also afforded the desired products in 93% and 95% yields, respectively. Notably, no product was isolated in case of glyoxal monoxime. The plausible reaction mechanism proposed by the authors proceeded through *cine*-substitution. In the first step, the activation of imidazole 1-oxide **22** by oxalyl chloride led to the generation of imidazolium chloride **24**, which transformed into intermediate **25** through nucleophilic substitution. Finally, the acidic hydrogen atom at C-2 position of the imidazole moiety was captured by triethylamine as base to provide the expected product **23**.

### Multicomponent reactions

**Scheme 6:**
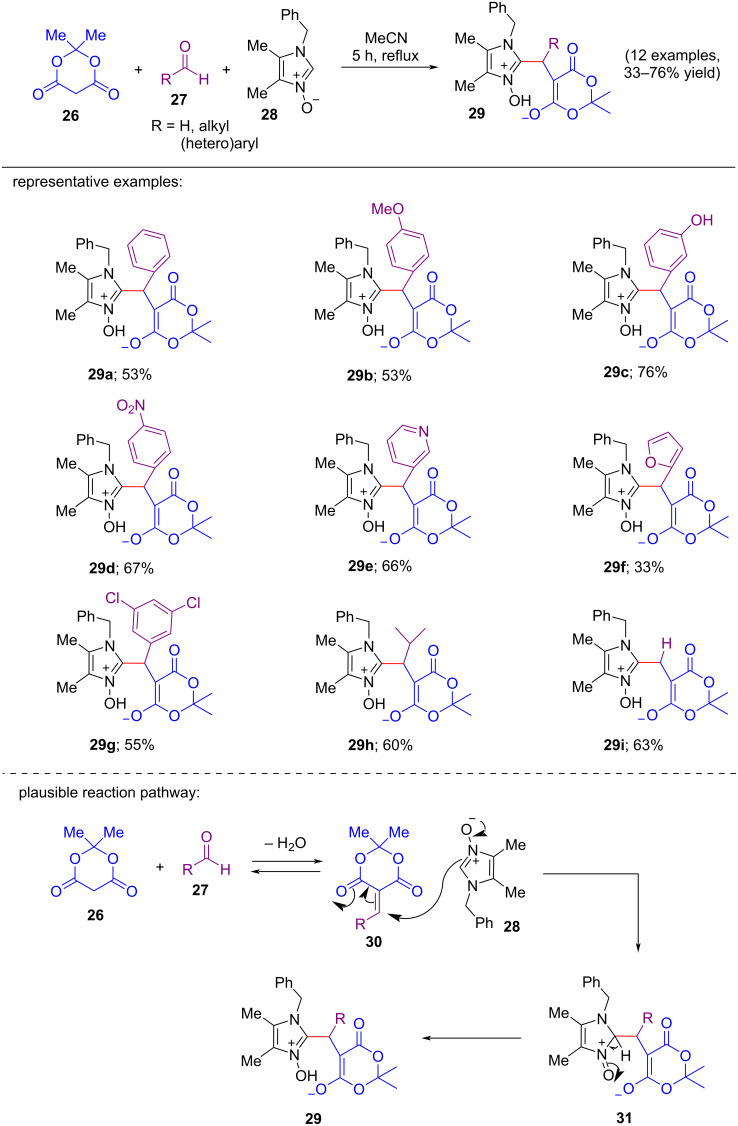
Multicomponent reaction of imidazole *N-*oxides **28** with Meldrum’s acid (**26**) and aldehydes.

In 2016, Vitaly S. Mityanov and co-workers unfolded a multicomponent condensation reaction methodology for the C-2 functionalization of 2-unsubstituted imidazole *N-*oxides using aldehydes and Meldrum’s acid under metal-free and mild reaction conditions [20]. The optimization conditions were estimated to be 1.0 mmol of 1-benzyl-4,5-dimethylimidazole *N-*oxide as substrate, 1.0 mmol of aldehydes and 1.0 mmol of Meldrum’s acid (**26**) in 6.0 mL of acetonitrile as solvent at reflux for 5 h. In this operationally simple procedure, the imidazole *N-*oxide plays the role of a ‘C’-nucleophile when there is no involvement of acid or base catalyst. 1-Benzyl-4,5-dimethylimidazole *N-*oxide (**28**) was chosen as the *N-*oxide substrate to react with Meldrum’s acid (**26**) and several aldehydes **27**. Here, heteroaromatic, aromatic, and aliphatic aldehydes along with formaldehyde were taken into consideration providing the corresponding products in 33–76% yield (Scheme 6). Based on the plausible mechanism proposed by the authors, most likely the reaction began with the nucleophilic addition reaction of Meldrum’s acid (**26**) and aldehyde **27** resulting in the formation of the electron-deficient enone **30**, which then participated in a Michael-type addition reaction with 1,3-dipolar 2-unsubstituted imidazole *N-*oxide **28** to provide the intermediate **31**. In the last step, the final product **29** was generated from the intermediate **31** with formation of the imidazole moiety.

**Scheme 7:**
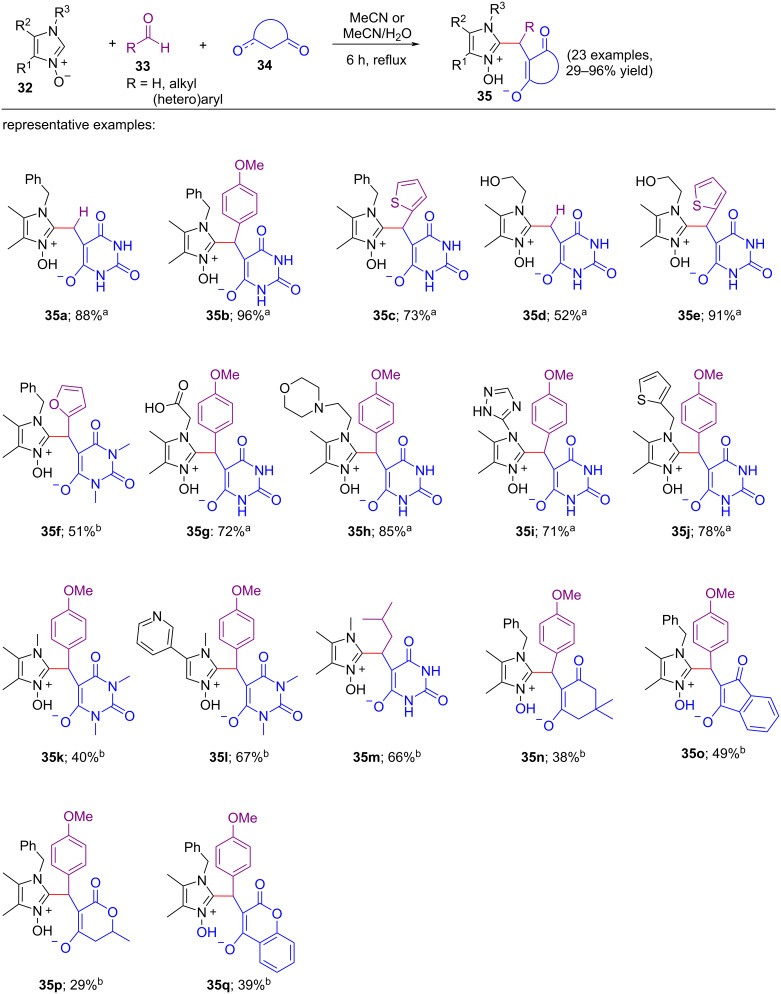
Multicomponent reaction of imidazole *N-*oxides with CH-acids and aldehydes. Reaction conditions: ^a^The mixture of 2-unsbstituted imidazole 1-oxide **32** (2 mmol), aldehyde **33** (2 mmol) and CH acid **34** (2 mmol, barbituric acid) was refluxed in H_2_O (2 mL) and MeCN (5 mL) solvent mixture for 8 h. ^b^The mixture of imidazole *N-*oxide **32** (2 mmol), CH-acid **34** (2 mmol, dimedone, 4-hydroxy-6-methyl-2*H*-pyran-2-one, 1,3-dimethylbarbituric acid, 4-hydroxycoumarin or 1,3-indandione), and aldehyde **33** (2 mmol) in MeCN (5 mL) as solvent was refluxed for 6 h.

In the next year (2017), the same authors reported another convenient metal-free multicomponent synthetic approach for the C-2 functionalization of 2-unsubstituted imidazole 1-oxides using CH-acids and aldehydes [21]. Here, the mixture of 2.0 mmol of imidazole *N-*oxides **32**, 2.0 mmol of aldehyde **33**, and 2.0 mmol of barbituric acid **34** was refluxed in the solvent mixture of 5.0 mL of MeCN and 2.0 mL of water for 6 h. Under the optimized conditions, the study of the substrate scope showed that the corresponding condensation products **35a–q** were obtained in 29–96% yields (Scheme 7). Various aldehydes, like heteroaromatic, aromatic, aliphatic, and formaldehyde as 37% solutions in water were chosen to react with several 1-arylimidazole *N*-oxides, 2-unsubstituted 1-alkyl-4,5-dimethylimidazole *N-*oxides, and 1-alkyl-2,4-unsubstituted imidazole 1-oxides. The selected CH-acids to study the scope of substrates were barbituric acid, dimedone, 4-hydroxy-6-methyl-2*H*-pyran-2-one, 1,3-dimethylbarbituric acid, 4-hydroxycoumarin, and 1,3-indandione. The nature of the CH-acids and 2-unsubstituted imidazole *N-*oxides did not affect the yield of products. Interestingly, unstable enones formed from the CH-acids like 4-hydroxy-6-methylpyrone and 4-hydroxycoumarin also successfully gave the desired products **35p** and **35q** under the standard conditions. The proposed reaction mechanism proceeded through the same pathway as outlined in Scheme 5. The reaction between the aldehyde and C–H acids followed by the attack of the imidazole *N-*oxides led to the generation of the final product.

**Scheme 8:**
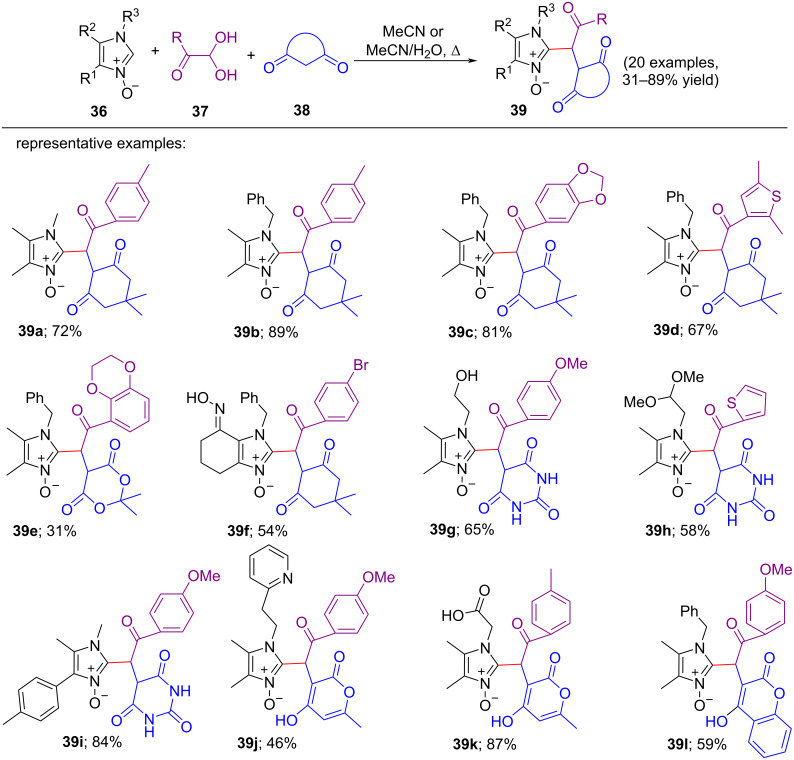
Three-component condensation reaction of imidazole *N-*oxides, arylglyoxals, and CH-acids **38** (dimedone, barbituric acid, Meldrum’s acid, 4-hydroxycoumarin, hydroxy-6-methylpyranone, or 4-hydroxy-7,7-dimethyl-7,8-dihydro-2*H*-chromene-2,5(6*H*)-dione).

In 2020, Valery P. Perevalov et al. introduced one more interesting simple and metal-free three-component condensation methodology for the C-2 functionalization reaction of 2-unsubstituted imidazole 1-oxides with arylglyoxals and CH-acids [11]. This procedure is convenient for the synthesis of pharmacologically important compounds containing furopyranone and furocoumarin scaffolds. With this method, extensive varieties of polyfunctional imidazole 1-oxides can be obtained. Here, 2-unsubstituted imidazole *N-*oxides were refluxed with several CH-acids and the heteroaromatic or aromatic hydrates of arylglyoxal in MeCN or a mixture of MeCN and water as solvent to give the corresponding condensation products in modest to good yields (31–89%) (Scheme 8). In this reported work, just akin to previously mentioned methodology in Scheme 7, a wide range of imidazole *N-*oxides and CH-acids like dimedone, barbituric acid, Meldrum’s acid, 4-hydroxycoumarin, hydroxy-6-methylpyranone, and 4-hydroxy-7,7-dimethyl-7,8-dihydro-2*H*-chromene-2,5(6*H*)-dione were chosen along with arylglyoxals to investigate the scope of substrates. No products were obtained in case of the acyclic CH-acids like dimethyl malonate, acetylacetone, and ethyl acetoacetate under the same conditions. The reaction follows almost the same mechanistic pathway that was previously mentioned as three-component reaction. However, in this reaction process, 2.0 equiv of CH-acids were needed against arylglyoxals since the electrophilic character of glyoxals is higher than that of aldehydes, generating less stable enones while reacting with CH-acids. First, 1.0 equiv CH-acid was reacted with 1.0 equiv arylglyoxal to generate an intermediate followed by immediate reaction with another 1.0 equiv CH-acids to form another reactive species which reacted with substituted imidazole *N-*oxide to produce desired products.

### Synthesis and trapping of optically active carbene

**Scheme 9:**
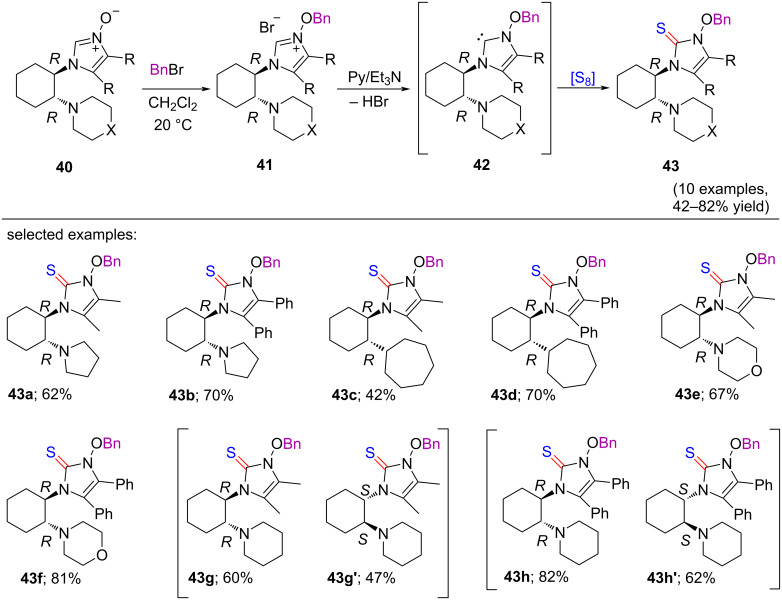
Synthesis of imidazole-2-thiones containing cyclohexyl-substituents at 3-position.

In 2019, Grzegorz Mlostoń and co-workers disclosed an excellent synthetic route for the preparation of chiral 3-alkoxyimidazol-2-ylidene intermediates [22]. Optically active 2-unsubstituted imidazole *N-*oxides were converted to carbene intermediates with retaining their stereochemistry. The appearance of the carbene intermediate was verified by trapping reactions with elemental sulfur [23–25], resulting in the generation of non-enolizable imidazole-2-thiones. At first, the alkylation of 2-unsubstituted imidazole *N-*oxides **40** took place in the presence of an equimolar quantity of benzyl bromide in CH_2_Cl_2_ at rt providing the (*N-*benzyloxy)imidazolium salts **41**. The latter underwent deprotonation in the presence of triethylamine in pyridine to generate the carbene intermediates **42** (Scheme 9). After that, the optically active imidazole-2-thiones **43** were obtained through the reaction with elemental sulfur. In CHCl_3_ solutions, the study of the optical rotation of the isolated products **43a–h** did not show any racemization under the standard reaction conditions.

**Scheme 10:**
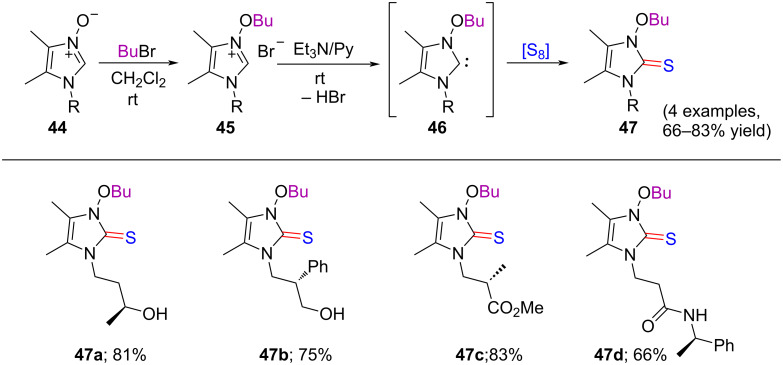
Preparation of optically active derivatives of 3-butoxyimidazole-2-thione.

Thereafter, imidazolium salts **45a–d**, obtained from imidazole *N-*oxides **44a–d**, provided the desired optically active 3-butoxyimidazole-2-thiones **47a–d** in 66–83% yield using triethylamine in pyridine followed by the treatment of elemental sulfur, through the formation of the optically active intermediate *N*-butoxyimidazol-2-ylidenes **46** (Scheme 10).

## Conclusion

Although there is a limited number of reports for synthetic approaches using imidazole *N-*oxides as substrates, the procedures are highly diverse. Transition-metal-free three-component reactions, cycloaddition reaction methodology and nucleophilic addition reaction, mainly these three types of reaction methodologies can be found in the literature reports. The approaches were greener and the reaction conditions were milder or not harsh in most of the cases and also the substrate scope was very wide. Imidazole *N-*oxides were functionalized at the C-2 and C-5 positions in the literature reports discussed in this review. However, no methodologies for the functionalization of imidazole *N-*oxide at the C-4 position were introduced till now. Also, C-5 functionalized products were generated only by the metal-free coupling reaction through nucleophilic substitution of H-atom (S_N_^H^). However, it can be noticed that these procedures were reported in the last 10 years and before there was no reported work regarding this research. So, it can be concluded that this is a new emerging research area and which offers a lot of room for further development. In this review, several research procedures were discussed with their substrate scope and proposed reaction mechanisms. We hope that this review can be helpful to the researchers and the chemists for the future improvements in this research area and that there will be growing interest to work in this domain of research. This would provide more exceptional and versatile literature reports with innovative research procedures to make this research field prosperous.
